# 4-Hy­droxy-3-meth­oxy­benzaldehyde–nicotinamide (1/1)

**DOI:** 10.1107/S1600536811045648

**Published:** 2011-11-05

**Authors:** Fiona N.-F. How, M. S. Amalina, Hamid Khaledi, Hapipah Mohd Ali

**Affiliations:** a Department of Biomedical Sciences, Kulliyah of Science, IIUM Kuantan, Jalan Sultan Ahmad Shah, Bandar Indera Mahkota, 25200 Kuantan, Pahang Darul Makmur, Malaysia; b Department of Chemistry, University of Malaya, 50603 Kuala Lumpur, Malaysia

## Abstract

In the title compound, C_6_H_6_N_2_O·C_8_H_8_O_3_, an equimolar co-crystal of nicotinamide and vanillin, the aromatic ring and the amide fragment of the nicotinamide mol­ecule make a dihedral angle of 32.6 (2)°. The vanillin mol­ecule is almost planar, with an r.m.s. deviation for all non-H atoms of 0.0094 Å. The vaniline and nicotinamide aromatic rings are nearly coplanar, the dihedral angle between them being 3.20 (9)°. In the crystal, the two components are linked through N—H⋯O and O—H⋯N hydrogen bonds into chains along the *a* axis. The chains are connected *via* C—H⋯O inter­actions, forming a three-dimensional polymeric structure.

## Related literature

For the crystal structure of nicotinamide, see: Miwa *et al.* (1999 ▶); Li *et al.* (2011 ▶). For the structure of vanillin, see: Velavan *et al.* (1995 ▶).
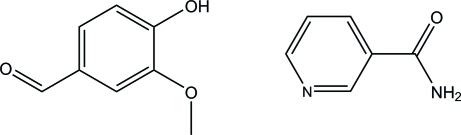


## Experimental

### 

#### Crystal data

C_6_H_6_N_2_O·C_8_H_8_O_3_
*M* *_r_* = 274.27
Triclinic, 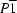

*a* = 4.8979 (1) Å
*b* = 8.5440 (2) Å
*c* = 15.4713 (4) Å
α = 98.108 (1)°
β = 92.810 (2)°
γ = 94.784 (2)°
*V* = 637.52 (3) Å^3^
*Z* = 2
Mo *K*α radiation
μ = 0.11 mm^−1^
*T* = 100 K
0.22 × 0.14 × 0.04 mm

#### Data collection

Bruker APEXII CCD diffractometer
Absorption correction: multi-scan (*SADABS*; Sheldrick, 1996 ▶) *T*_min_ = 0.977, *T*_max_ = 0.996
3432 measured reflections
2243 independent reflections
1862 reflections with *I* > 2σ(*I*)
*R*_int_ = 0.019

#### Refinement

*R*[*F* ^2^ > 2σ(*F* ^2^)] = 0.045
*wR*(*F* ^2^) = 0.112
*S* = 1.05
2243 reflections
191 parameters
4 restraints
H atoms treated by a mixture of independent and constrained refinement
Δρ_max_ = 0.66 e Å^−3^
Δρ_min_ = −0.28 e Å^−3^

### 

Data collection: *APEX2* (Bruker, 2007 ▶); cell refinement: *SAINT* (Bruker, 2007 ▶); data reduction: *SAINT*; program(s) used to solve structure: *SHELXS97* (Sheldrick, 2008 ▶); program(s) used to refine structure: *SHELXL97* (Sheldrick, 2008 ▶); molecular graphics: *X-SEED* (Barbour, 2001 ▶); software used to prepare material for publication: *SHELXL97* and *publCIF* (Westrip, 2010 ▶).

## Supplementary Material

Crystal structure: contains datablock(s) I, global. DOI: 10.1107/S1600536811045648/pv2471sup1.cif

Structure factors: contains datablock(s) I. DOI: 10.1107/S1600536811045648/pv2471Isup2.hkl

Supplementary material file. DOI: 10.1107/S1600536811045648/pv2471Isup3.cml

Additional supplementary materials:  crystallographic information; 3D view; checkCIF report

## Figures and Tables

**Table 1:** Hydrogen-bond geometry (Å, °)

*D*—H⋯*A*	*D*—H	H⋯*A*	*D*⋯*A*	*D*—H⋯*A*
N1—H1*A*⋯O4^i^	0.87 (2)	2.05 (2)	2.900 (2)	167 (2)
N1—H1*B*⋯O2^ii^	0.87 (2)	2.42 (2)	3.085 (2)	134 (2)
N1—H1*B*⋯O3^ii^	0.87 (2)	2.20 (2)	3.019 (2)	156 (2)
O2—H2⋯N2^iii^	0.85 (2)	1.80 (2)	2.634 (2)	164 (2)
C8—H8*A*⋯O1^iv^	0.98	2.59	3.381 (3)	137
C8—H8*C*⋯O2^i^	0.98	2.55	3.337 (2)	138
C13—H13⋯O1^v^	0.95	2.49	3.185 (3)	130

Symmetry codes: (i) 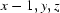; (ii) 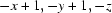; (iii) 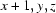; (iv) 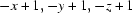; (v) 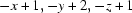.

**Fig. 1.**
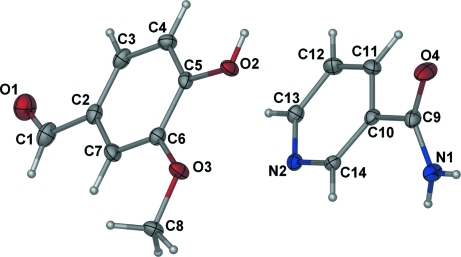
Molecular structure of the title compound with displacement ellipsoids drawn at 50% probability level. Hydrogen atoms are drawn as spheres of arbitrary radii.

**Fig. 2.**
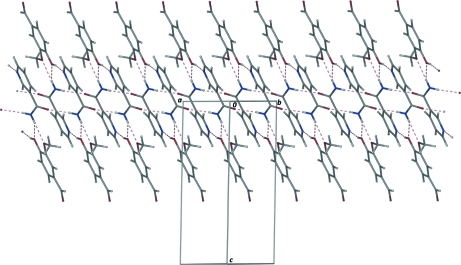
A chain along the a axis formed by N—H···O and O—H···N hydrogen bonds.

## supplementary crystallographic information

### Comment

The crystal structures of nicotinamide (Miwa *et al.*,1999; Li *et al.*, 2011) and 4-hydroxy-3-methoxybenzaldehyde, vanillin, (Velavan *et al.*,1995) have been previously reported. The title compound is an equimolar cocrystal of nicotinamide and vanillin (Fig. 1). The nicotinamide aromatic ring and the plane of the amide fragment, N1—C9—O4, are twisted with respect to each other, making a dihedral angle of 32.6 (2)°. The vanillin molecule is essentially planar, the highest deviation from the best plane passing through all non-H atoms being 0.0156 (13) Å for O3 atom. In the crystal, the molecules of nicotinamide and vanillin are linked through N—H···O and O—H···N hydrogen bonds into infinite chains along the *a* axis (Fig. 2). The chains are connected *via* C—H···O interactions (Table 1 and Fig. 2) to form a three-dimensional polymeric structure.

### Experimental

A mixture of vanillin (1.52 g, 0.1 mol) and nicotinamide (1.22 g, 0.1 mol) in ethanol (30 ml) was heated for 1 hr. The solvent was then evaporated partially and the solution was left at room temperature. The colorless crystals of the title compound were obtained in a day.

### Refinement

The C-bound H atoms were placed at calculated positions and were treated as riding on their parent C atoms with C—H distances of 0.95 (aryl) and 0.98 (methyl) Å. The N– and O-bound H atoms were located in a difference Fourier map, and refined with distance restraints of O—H = 0.84 (2) Å and N—H = 0.88 (2) Å. For all H atoms, *U*_iso_(H) was set to 1.2–1.5_eq_(carrier atom). An additional rigid-bond type restraint (*DELU* in *SHELXL97*) was placed on the displacement parameters of C1 and C2.

### Crystal data

**Table:**

C_6_H_6_N_2_O·C_8_H_8_O_3_	*Z* = 2
*M**_r_* = 274.27	*F*(000) = 288
Triclinic, *P*1	*D*_x_ = 1.429 Mg m^−^^3^
Hall symbol: -P 1	Mo *K*α radiation, λ = 0.71073 Å
*a* = 4.8979 (1) Å	Cell parameters from 1226 reflections
*b* = 8.5440 (2) Å	θ = 2.6–29.7°
*c* = 15.4713 (4) Å	µ = 0.11 mm^−^^1^
α = 98.108 (1)°	*T* = 100 K
β = 92.810 (2)°	Lath, colorless
γ = 94.784 (2)°	0.22 × 0.14 × 0.04 mm
*V* = 637.52 (3) Å^3^	

### Data collection

**Table:**

Bruker APEXII CCD diffractometer	2243 independent reflections
Radiation source: fine-focus sealed tube	1862 reflections with *I* > 2σ(*I*)
graphite	*R*_int_ = 0.019
φ and ω scans	θ_max_ = 25.3°, θ_min_ = 2.4°
Absorption correction: multi-scan (*SADABS*; Sheldrick, 1996)	*h* = −5→5
*T*_min_ = 0.977, *T*_max_ = 0.996	*k* = −10→10
3432 measured reflections	*l* = −18→18

### Refinement

**Table:**

Refinement on *F*^2^	Primary atom site location: structure-invariant direct methods
Least-squares matrix: full	Secondary atom site location: difference Fourier map
*R*[*F*^2^ > 2σ(*F*^2^)] = 0.045	Hydrogen site location: inferred from neighbouring sites
*wR*(*F*^2^) = 0.112	H atoms treated by a mixture of independent and constrained refinement
*S* = 1.05	*w* = 1/[σ^2^(*F*_o_^2^) + (0.0439*P*)^2^ + 0.4685*P*] where *P* = (*F*_o_^2^ + 2*F*_c_^2^)/3
2243 reflections	(Δ/σ)_max_ < 0.001
191 parameters	Δρ_max_ = 0.66 e Å^−^^3^
4 restraints	Δρ_min_ = −0.28 e Å^−^^3^

### Special details

**Table:**

Geometry. All e.s.d.'s (except the e.s.d. in the dihedral angle between two l.s. planes) are estimated using the full covariance matrix. The cell e.s.d.'s are taken into account individually in the estimation of e.s.d.'s in distances, angles and torsion angles; correlations between e.s.d.'s in cell parameters are only used when they are defined by crystal symmetry. An approximate (isotropic) treatment of cell e.s.d.'s is used for estimating e.s.d.'s involving l.s. planes.
Refinement. Refinement of *F*^2^ against ALL reflections. The weighted *R*-factor *wR* and goodness of fit *S* are based on *F*^2^, conventional *R*-factors *R* are based on *F*, with *F* set to zero for negative *F*^2^. The threshold expression of *F*^2^ > σ(*F*^2^) is used only for calculating *R*-factors(gt) *etc*. and is not relevant to the choice of reflections for refinement. *R*-factors based on *F*^2^ are statistically about twice as large as those based on *F*, and *R*- factors based on ALL data will be even larger.

### Fractional atomic coordinates and isotropic or equivalent isotropic displacement parameters (Å^2^)

**Table:**

	*x*	*y*	*z*	*U*_iso_*/*U*_eq_	
O1	0.3651 (3)	0.83011 (19)	0.61085 (10)	0.0359 (4)	
O2	0.8107 (3)	0.63328 (16)	0.23075 (9)	0.0195 (3)	
H2	0.949 (4)	0.698 (2)	0.2250 (15)	0.029*	
O3	0.4028 (3)	0.44026 (16)	0.25734 (9)	0.0218 (3)	
C1	0.3070 (5)	0.7306 (3)	0.54863 (14)	0.0286 (5)	
H1	0.1529	0.6570	0.5522	0.034*	
C2	0.4516 (4)	0.7113 (2)	0.46701 (13)	0.0210 (4)	
C3	0.6726 (4)	0.8148 (2)	0.45164 (13)	0.0221 (5)	
H3	0.7380	0.9016	0.4951	0.026*	
C4	0.7972 (4)	0.7912 (2)	0.37307 (13)	0.0204 (4)	
H4	0.9482	0.8620	0.3629	0.024*	
C5	0.7035 (4)	0.6648 (2)	0.30888 (12)	0.0166 (4)	
C6	0.4793 (4)	0.5600 (2)	0.32443 (12)	0.0176 (4)	
C7	0.3561 (4)	0.5847 (2)	0.40255 (13)	0.0208 (5)	
H7	0.2040	0.5146	0.4128	0.025*	
C8	0.1709 (4)	0.3323 (2)	0.26827 (14)	0.0217 (5)	
H8A	0.2110	0.2765	0.3179	0.033*	
H8B	0.1328	0.2551	0.2150	0.033*	
H8C	0.0103	0.3917	0.2793	0.033*	
O4	0.8254 (3)	0.71964 (19)	−0.04761 (9)	0.0299 (4)	
N1	0.3766 (4)	0.6341 (2)	−0.07875 (11)	0.0205 (4)	
H1A	0.207 (3)	0.645 (3)	−0.0672 (14)	0.025*	
H1B	0.415 (4)	0.586 (2)	−0.1293 (11)	0.025*	
N2	0.2385 (3)	0.80224 (19)	0.18395 (10)	0.0170 (4)	
C9	0.5840 (4)	0.7129 (2)	−0.02755 (13)	0.0197 (4)	
C10	0.5123 (4)	0.7959 (2)	0.05932 (12)	0.0169 (4)	
C11	0.6653 (4)	0.9350 (2)	0.09779 (13)	0.0201 (4)	
H11	0.8121	0.9801	0.0687	0.024*	
C12	0.5998 (4)	1.0066 (2)	0.17913 (13)	0.0220 (5)	
H12	0.6997	1.1023	0.2066	0.026*	
C13	0.3864 (4)	0.9363 (2)	0.21971 (13)	0.0192 (4)	
H13	0.3429	0.9856	0.2757	0.023*	
C14	0.3015 (4)	0.7346 (2)	0.10499 (12)	0.0162 (4)	
H14	0.1966	0.6397	0.0788	0.019*	

### Atomic displacement parameters (Å^2^)

**Table:**

	*U*^11^	*U*^22^	*U*^33^	*U*^12^	*U*^13^	*U*^23^
O1	0.0440 (10)	0.0363 (9)	0.0266 (9)	0.0056 (8)	0.0038 (7)	0.0006 (7)
O2	0.0182 (8)	0.0217 (7)	0.0174 (7)	−0.0031 (6)	0.0053 (6)	0.0004 (6)
O3	0.0223 (8)	0.0214 (7)	0.0198 (7)	−0.0045 (6)	0.0064 (6)	−0.0016 (6)
C1	0.0356 (13)	0.0308 (12)	0.0189 (10)	0.0104 (10)	−0.0028 (9)	−0.0012 (9)
C2	0.0226 (11)	0.0246 (11)	0.0170 (10)	0.0074 (8)	0.0004 (8)	0.0045 (8)
C3	0.0270 (11)	0.0214 (10)	0.0165 (10)	0.0060 (9)	−0.0036 (8)	−0.0020 (8)
C4	0.0187 (10)	0.0193 (10)	0.0218 (11)	−0.0018 (8)	−0.0001 (8)	0.0014 (8)
C5	0.0157 (10)	0.0209 (10)	0.0145 (9)	0.0054 (8)	0.0026 (8)	0.0043 (8)
C6	0.0193 (10)	0.0176 (10)	0.0157 (10)	0.0033 (8)	0.0002 (8)	0.0007 (8)
C7	0.0193 (11)	0.0242 (11)	0.0199 (10)	0.0020 (8)	0.0046 (8)	0.0055 (8)
C8	0.0185 (11)	0.0198 (10)	0.0259 (11)	−0.0031 (8)	0.0035 (8)	0.0021 (8)
O4	0.0144 (8)	0.0516 (10)	0.0228 (8)	0.0064 (7)	0.0038 (6)	−0.0003 (7)
N1	0.0172 (9)	0.0279 (9)	0.0153 (9)	0.0046 (7)	0.0035 (7)	−0.0030 (7)
N2	0.0171 (9)	0.0194 (8)	0.0148 (8)	0.0031 (7)	0.0012 (6)	0.0027 (6)
C9	0.0178 (11)	0.0255 (11)	0.0168 (10)	0.0055 (8)	0.0018 (8)	0.0042 (8)
C10	0.0144 (10)	0.0210 (10)	0.0156 (10)	0.0038 (8)	−0.0008 (7)	0.0030 (8)
C11	0.0149 (10)	0.0240 (10)	0.0221 (10)	0.0003 (8)	0.0020 (8)	0.0058 (8)
C12	0.0227 (11)	0.0180 (10)	0.0234 (11)	−0.0030 (8)	−0.0011 (8)	−0.0002 (8)
C13	0.0217 (11)	0.0192 (10)	0.0160 (10)	0.0031 (8)	0.0002 (8)	0.0001 (8)
C14	0.0157 (10)	0.0162 (9)	0.0161 (10)	0.0009 (7)	−0.0013 (8)	0.0012 (7)

### Geometric parameters (Å, °)

**Table:**

O1—C1	1.197 (3)	C8—H8B	0.9800
O2—C5	1.343 (2)	C8—H8C	0.9800
O2—H2	0.854 (16)	O4—C9	1.236 (2)
O3—C6	1.365 (2)	N1—C9	1.330 (3)
O3—C8	1.435 (2)	N1—H1A	0.868 (16)
C1—C2	1.474 (3)	N1—H1B	0.869 (16)
C1—H1	0.9500	N2—C13	1.336 (2)
C2—C3	1.391 (3)	N2—C14	1.338 (2)
C2—C7	1.396 (3)	C9—C10	1.500 (3)
C3—C4	1.384 (3)	C10—C14	1.388 (3)
C3—H3	0.9500	C10—C11	1.392 (3)
C4—C5	1.391 (3)	C11—C12	1.384 (3)
C4—H4	0.9500	C11—H11	0.9500
C5—C6	1.410 (3)	C12—C13	1.384 (3)
C6—C7	1.375 (3)	C12—H12	0.9500
C7—H7	0.9500	C13—H13	0.9500
C8—H8A	0.9800	C14—H14	0.9500
			
C5—O2—H2	112.6 (16)	O3—C8—H8C	109.5
C6—O3—C8	117.44 (15)	H8A—C8—H8C	109.5
O1—C1—C2	126.2 (2)	H8B—C8—H8C	109.5
O1—C1—H1	116.9	C9—N1—H1A	121.6 (15)
C2—C1—H1	116.9	C9—N1—H1B	117.5 (15)
C3—C2—C7	119.56 (18)	H1A—N1—H1B	120 (2)
C3—C2—C1	122.83 (19)	C13—N2—C14	117.72 (17)
C7—C2—C1	117.59 (19)	O4—C9—N1	123.82 (19)
C4—C3—C2	119.93 (19)	O4—C9—C10	119.84 (18)
C4—C3—H3	120.0	N1—C9—C10	116.34 (17)
C2—C3—H3	120.0	C14—C10—C11	118.15 (18)
C3—C4—C5	120.68 (18)	C14—C10—C9	121.73 (18)
C3—C4—H4	119.7	C11—C10—C9	120.07 (18)
C5—C4—H4	119.7	C12—C11—C10	118.88 (18)
O2—C5—C4	124.72 (18)	C12—C11—H11	120.6
O2—C5—C6	115.90 (17)	C10—C11—H11	120.6
C4—C5—C6	119.38 (18)	C13—C12—C11	118.77 (19)
O3—C6—C7	125.62 (18)	C13—C12—H12	120.6
O3—C6—C5	114.82 (17)	C11—C12—H12	120.6
C7—C6—C5	119.56 (18)	N2—C13—C12	123.12 (18)
C6—C7—C2	120.88 (19)	N2—C13—H13	118.4
C6—C7—H7	119.6	C12—C13—H13	118.4
C2—C7—H7	119.6	N2—C14—C10	123.34 (18)
O3—C8—H8A	109.5	N2—C14—H14	118.3
O3—C8—H8B	109.5	C10—C14—H14	118.3
H8A—C8—H8B	109.5		

### Hydrogen-bond geometry (Å, °)

**Table:**

*D*—H···*A*	*D*—H	H···*A*	*D*···*A*	*D*—H···*A*
N1—H1A···O4^i^	0.87 (2)	2.05 (2)	2.900 (2)	167 (2)
N1—H1B···O2^ii^	0.87 (2)	2.42 (2)	3.085 (2)	134.(2)
N1—H1B···O3^ii^	0.87 (2)	2.20 (2)	3.019 (2)	156 (2)
O2—H2···N2^iii^	0.85 (2)	1.80 (2)	2.634 (2)	164 (2)
C8—H8A···O1^iv^	0.98	2.59	3.381 (3)	137.
C8—H8C···O2^i^	0.98	2.55	3.337 (2)	138.
C13—H13···O1^v^	0.95	2.49	3.185 (3)	130.

Symmetry codes: (i) *x*−1, *y*, *z*; (ii) −*x*+1, −*y*+1, −*z*; (iii) *x*+1, *y*, *z*; (iv) −*x*+1, −*y*+1, −*z*+1; (v) −*x*+1, −*y*+2, −*z*+1.
